# Escaping the Phagocytic Oxidative Burst: The Role of SODB in the Survival of *Pseudomonas aeruginosa* Within Macrophages

**DOI:** 10.3389/fmicb.2020.00326

**Published:** 2020-03-10

**Authors:** Luca Cavinato, Elena Genise, Francesco R. Luly, Enea G. Di Domenico, Paola Del Porto, Fiorentina Ascenzioni

**Affiliations:** ^1^Department of Biology and Biotechnology “Charles Darwin”, Sapienza University of Rome, Rome, Italy; ^2^Microbiology and Virology, San Gallicano Dermatologic Institute, IRCCS, Rome, Italy

**Keywords:** *P. aeruginosa*, SODs, ROS, macrophages, autophagy, oxidative burst

## Abstract

Reactive oxygen species (ROS) are small oxygen-derived molecules that are used to control infections by phagocytic cells. In macrophages, the oxidative burst produced by the NOX2 NADPH-oxidase is essential to eradicate engulfed pathogens by both oxidative and non-oxidative killing. Indeed, while the superoxide anion (${\text{O}}_{2}^{-}$) produced by NOX2, and the other ROS derived from its transformation, can directly target pathogens, ROS also contribute to activation of non-oxidative microbicidal effectors. The response of pathogens to the phagocytic oxidative burst includes the expression of different enzymes that target ROS to reduce their toxicity. Superoxide dismutases (SODs) are the primary scavengers of ${\text{O}}_{2}^{-}$, which is transformed into H_2_O_2_. In the Gram-negative *Salmonella typhimurium*, periplasmic SODCI has a major role in bacterial resistance to NOX-mediated oxidative stress. In *Pseudomonas aeruginosa*, the two periplasmic SODs, SODB, and SODM, appear to contribute to bacterial virulence in small-animal models. Furthermore, NOX2 oxidative stress is essential to restrict *P. aeruginosa* survival in macrophages early after infection. Here, we focused on the role of *P. aeruginosa* SODs in the counteracting of the lethal effects of the macrophage oxidative burst. Through this study of the survival of *sod* mutants in macrophages and the measurement of ROS in infected macrophages, we have identified a dual, antagonistic, role for SODB in *P. aeruginosa* survival. Indeed, the survival of the *sodB* mutants, but not of the *sodM* mutants, was greater than that of the wild-type (WT) bacteria early after infection, and *sodB*-infected macrophages showed higher levels of ${\text{O}}_{2}^{-}$ and lower levels of H_2_O_2_. This suggests that SODB contributes to the production of lethal doses of H_2_O_2_ within the phagosome. However, later on following infection, the *sodB* mutants survived less that the WT bacteria, which highlights the pro-survival role of SODB. We have explained this defensive role through an investigation of the activation of autophagy, which was greater in the *sodB*-infected macrophages.

## Introduction

Macrophages are professional phagocytes, and their major role in the control of infectious diseases is the engulfment of microorganisms within phagosomes, which in a complex maturation process acquire disparate microbicidal effectors (Flannagan et al., 2009, 2012). One of the stronger and earlier microbicidal mechanisms is undoubtedly the production of reactive oxygen species (ROS), which are generated by the NOX2 NADPH oxidase (Lam et al., 2010; Panday et al., 2015). Following the engulfment of pathogens by macrophages, the NOX2 multisubunit complex is assembled and activated at the phagosome membrane, where it liberates superoxide anions, ${\text{O}}_{2}^{-}$, into the phagosomal lumen (Lam et al., 2010). In the phagosome, ${\text{O}}_{2}^{-}$ can be spontaneously or enzymatically dismutated to H_2_O_2_, which might, in turn, be converted to hydroxyl radicals (HO^–^) via the Fenton reaction. Importantly, the overall levels of H_2_O_2_ in phagosomes also depends on H_2_O_2_ leakage, which is favored by its high stability and neutral charge.

Phagocytic ROS might directly kill the engulfed pathogens or be transformed into other antimicrobial effectors. For example, myeloperoxidase targets H_2_O_2_ to produce hypochlorous acid, which has potent microbicidal activity (Fang, 2011). However, myeloperoxidase is mainly active in neutrophils, and it does not appear to be essential for host defense (Lanza, 1998; Klebanoff, 2005; Klebanoff et al., 2013). ROS cytotoxicity might also be enhanced by their interactions with other cellular mediators, such as NO (Fang, 2011). Pathogens enclosed within phagosomes are exposed to high levels of ROS, which are produced in their close proximity, and which can directly kill the engulfed bacteria by targeting different microbial macromolecules, such as their DNA and proteins, and in particular, iron–sulfur-clustered protein (Fang, 2011). The oxidative burst response follows specific temporal dynamics that impose high oxidative stress on the engulfed pathogens soon after infection, followed by extended periods of little, if any, ROS production. Therefore, the survival of pathogens in this environment is critically dependent on their detoxification of ROS in the early stages of an infection.

The microbial defenses against ROS include catalases, peroxidases, and superoxide dismutases (SODs) (Mishra and Imlay, 2012). Catalases and peroxidases target H_2_O_2_, which produces H_2_O and O_2_, while SODs promote dismutation of ${\text{O}}_{2}^{-}$ into H_2_O_2_ (Mishra and Imlay, 2012). Therefore, only the combined actions of these enzymes can directly detoxify the superoxide anions produced by the phagocytic NOX2. Additionally, by reducing the overall levels of superoxide radicals (1 mol ${\text{O}}_{2}^{-}$ per 0.5 mol H_2_O_2_), the SOD activity limits the possible toxicity of ${\text{O}}_{2}^{-}$ in this compartment (Craig and Slauch, 2009) and in the production of other cytotoxic mediators (Fang, 2011). In Gram-negative bacteria, ROS scavenger enzymes can occupy different subcellular compartments, with SODs localized to the periplasm, while catalase and peroxidase are mainly confined to the cytoplasm. This compartmentalization is functional for the targeting of ${\text{O}}_{2}^{-}$, the leakage of which is highly restricted by the negative charge in the periplasm and H_2_O_2_ in the cytoplasm (Aussel et al., 2011; Fang, 2011). The complexity of the anti-ROS defenses is further increased by the presence of more than one gene for each class of ROS-scavenger enzyme that encodes enzymes that differ in cofactor requirements, regulation, and sub-cellular localization (Fang, 2011; Imlay, 2019; Johnson and Hug, 2019).

Gram-negative bacteria entrapped within phagosomes can be exposed to rapid increases in the levels of superoxide radicals, which can be targeted by the periplasmic SOD. A major role of this periplasmic SOD to ensure bacterial survival in such environments was demonstrated in *Salmonella*. Indeed, the periplasmic SOD of *Salmonella*, SODCI, was predicted to be the only enzyme that critically impacts upon *Salmonella* survival in the phagosome (De Groote et al., 1997; Craig and Slauch, 2009), whereby in its absence, the ${\text{O}}_{2}^{-}$ levels can increase to lethal doses (Burton et al., 2014; Fenlon and Slauch, 2014). Additionally, in murine *in vivo* models, it was shown that *Salmonella* infection is controlled by NADPH-dependent oxidative killing, although this only refers to neutrophils, as in macrophages the oxidative burst appears to be sublethal for bacterial cells (Burton et al., 2014).

*Pseudomonas aeruginosa* is a Gram-negative facultative aerobic opportunistic pathogen that causes infections in humans, and particularly in immunocompromised patients and patients with cystic fibrosis (CF) (Ciofu et al., 2015; Klockgether and Tümmler, 2017). In CF, *P. aeruginosa* intermittently infects the airways of children and young patients, although as the age of the patient increases, *P. aeruginosa* can stably colonize the CF lungs, and represents the major cause of pulmonary disease while contributing to the morbidity and mortality of patients with CF (Elborn, 2016; Malhotra et al., 2019). The CF lungs are dominated by high levels of ROS, which result from chronic bacterial infections, large inflammatory responses, and defective antioxidant production, such as glutathione (Galli et al., 2012; de Bari et al., 2018; Malhotra et al., 2019). This scenario clearly suggests that the ROS defense of *P. aeruginosa* must be decisively important for colonization of the CF lungs. This colonization has been assigned in part to the impaired activity of innate immune cells, which do not appear to eradicate infections (Bruscia and Bonfield, 2016). Accordingly, we and others have demonstrated that the microbicidal activity against *P. aeruginosa* of CF macrophages is impaired (Del Porto et al., 2011; Simonin-Le Jeune et al., 2013). However, we have shown that the oxidative burst of human CF macrophages is similar to that of non-CF cells, and is functional to kill *P. aeruginosa* early after infection (Cifani et al., 2013).

Thus, to better understand how *P. aeruginosa* tackles this oxidative killing by the macrophages, we investigated the role of the bacterial SODs, which are the first enzymatic activities involved in superoxide radical detoxification. *P. aeruginosa* SODs have been shown previously to contribute to bacterial virulence in the small-animal model of the silkmoth *Bombyx mori*, the mortality of which was reduced by infection with *P. aeruginosa sod* mutants, with respect to wild-type (WT) strains (Iiyama et al., 2007). Furthermore, analysis of *sodB* and *sodM* mutants has showed that for *P. aeruginosa* virulence, SODB is more critical than SODM. SODB is an iron-cofactored SOD (Fe-SOD), while SODM requires manganese (Mn-SOD), and both localize to the periplasm (Winsor et al., 2016). The expression of these SODs is regulated by the availability of the iron and manganese cofactors and specific environmental conditions, although SODB is highly expressed under all growth conditions, while SODM is restricted to low-Fe requirements (Hassett et al., 1993, 1995).

Here, by analyzing the survival of the *P. aeruginosa* WT and *sod* mutant strains in murine and human macrophages, we demonstrate a dual antagonistic role of *P. aeruginosa* SODB, which modulates the microbicidal activity of macrophages by acting first as a pro-microbicidal and then as a pro-survival.

## Materials and Methods

### Macrophages, Media, and Supplements

RAW246.7 murine macrophages (ATCC, TIB71) were cultivated in Dulbecco’s modified Eagle’s medium (DMEM; Corning, United States) supplemented with 10% fetal bovine serum, 1 mM glutamine, 100 U/μL penicillin and 100 μg/mL streptomycin (all from EuroClone, Italy). The cells were grown in tissue culture flasks or multiwell plates, at 37°C and 5% CO_2_. Human monocyte-derived macrophages (HMDMs) were differentiated *in vitro* from monocytes isolated from the buffy coats of healthy donors, as previously described (Del Porto et al., 2011). Briefly, peripheral blood mononuclear cells were isolated by density gradient centrifugation (Lympholyte; Cedarlane, Hornby, CA, United States), and were selected with an anti-CD14 monoclonal antibody coupled to magnetic beads (Miltenyi Biotec, Bergisch Gladbach, Germany). The CD14^+^ cells were differentiated for 7 days in Roswell Park Memorial Institute (RPMI) 1640 (Gibco-BRL, Invitrogen Corporation, Carlsbad, CA, United States) supplemented with 20% fetal bovine serum and 100 ng/mL recombinant macrophage colony stimulating factor (PeproTech Inch, Rocky Hill, NY, United States).

### Bacterial Strains and Growth Conditions

The bacterial strains and plasmid used in this study are listed in Table 1. The *P. aeruginosa* mutant strains were recovered from frozen stocks and analyzed for the gene deletions. Genomic DNA extraction and gene amplification were performed as previously reported (Di Domenico et al., 2015), using primers listed in Supplementary Table S1. As expected, sodB and sodM amplification bands were detected in the *P. aeruginosa* WT (PAO1). In contrast, sodB and sodM amplification bands were absent in the *sodB* and *sodM* mutants, respectively (Supplementary Figure S1). All of these strains were grown from single colonies in Luria-Bertani (LB) medium (Sigma, United States) at 30°C, with liquid cultures grown with shaking at 180 rpm. The *sodB* and *sodM* mutant strains were routinely grown in LB medium containing 50 μg/mL tetracycline and gentamycin, respectively. As expected from previous studies (Hassett et al., 1995; Iiyama et al., 2007), the *sodB* mutant grew more slowly than the parental PAO1 strain (Supplementary Figure S2). The *P. aeruginosa* strains that expressed green fluorescent protein (GFP) were obtained by electroporation of pUC30T-*gfpmut*3 (Barbier and Damron, 2016) and selection in 15 μg/mL gentamycin on LB agar plates.

**TABLE 1:** Bacterial strains and plasmid used in this study.

Name	Description^a^	References
PAO1	Wild-type strain	Iiyama et al., 2007
PAO1*sodB*	PAO1 *sodB* mutant; *sodB*:Ω*Tc*, Tc ^r^	Iiyama et al., 2007
PAO1*sodM*	PAO1 *sodM* mutant; *sodM*:Ω*aac*, Gm ^r^	Iiyama et al., 2007
pUC30T-*gfpmut*3	Plasmid encoding GFP, Gm^r^	Barbier and Damron, 2016

*^*a*^Tc^*r*^, tetracycline resistance; Gm^*r*^, gentamicin resistance; GFP, green fluorescence protein.*

### *P. aeruginosa* Killing by Macrophages

The day before infection, the macrophages were seeded into 48-well plates (10^5^ cells/well) in culture medium without antibiotics, and incubated at 37°C in 5% CO_2_. When applied, the macrophages were pretreated (i.e., before infection) with the NADPH oxidase inhibitor, diphenyleneiodonium (DPI; Sigma, United States), at 10 μM for 30 min. Exponentially growing *P. aeruginosa* cells were prepared by refreshing the overnight cultures in LB broth at 30°C (Supplementary Material). After two washes in phosphate-buffered saline (PBS), the *P. aeruginosa* were resuspended in antibiotic-free cell-culture medium and added to the macrophages at a multiplicity of infection (MOI) of 10. The infection was synchronized by centrifugation of the multiwell plates (550 × *g* for 5 min), which were then incubated at 37°C in 5% CO_2_ for 30 min to 60 min. At the end of the infection, the cells were washed with PBS and incubated in DMEM containing 1 mg/mL amikacin and 1 mg/mL ceftazidime for 15 min. Afterward, the macrophages in selected wells (defined as *t*_0_) were lysed in 1% Triton X-100 for 10 min at room temperature, and finally diluted to 1 mL PBS. The cell viability was determined according to the colony-forming unit (CFU) assay. In the remaining wells, the medium was replaced with culture medium supplemented with a sub-inhibitory concentration of the antibiotics (0.1 mg/mL amikacin; 0.1 mg/mL ceftazidime) and incubated for a further 60 min (*t*_60_) or 180 min (*t*_180_). At the end of the incubations, the live *P. aeruginosa* were recovered as described above. The bacteria survival was calculated according to Eq. 1:

(1) $$100-\left\{\left[\left({\text{CFU}}_{t_{0}}-{\text{CFU}}_{t_{180}}\right)\,/\,{\text{CFU}}_{t_{0}}\right]\times 100\right\}$$

### Phagocytosis Assays

The macrophages (i.e., RAW 264.7 cells, HMDMs) were seeded in 24-well plates (2 × 10^5^ cells/well) in antibiotics-free medium the day before infection, and then infected with GFP-expressing PAO1 and PAO1 *sodB P. aeruginosa* strains, at a MOI of 25. Phagocytosis was carried out by incubation of the infected macrophages for 30 min or 60 min at 37°C in 5% CO_2_. Afterward, the cells were gently washed two or three times with PBS, enzymatically detached, and analyzed by flow cytometry (BD FACSCalibur, France). Phagocytosis was evaluated as the fraction of GFP^+^ cells in the bulk population. The data were analyzed using the CellQuest software, and the images were processed with FlowJo.

### ROS Measurements

Intracellular ${\text{O}}_{2}^{-}$ levels were measured using luminol (Sigma, United States). Briefly, the macrophages were resuspended in Hank’s balanced salt solution (HBSS) without phenol red (Sigma, United States), supplemented with 25 μg/mL luminol, and seeded in white 96-well plates (Sarstedt, Germany). The RAW264.7 macrophages were seeded at 3 × 10^5^ cells/well, with the HMDMs at 10^5^ cells/well. The macrophages were challenged with the *P. aeruginosa* strains at a MOI of 10, and the chemiluminescence was measured at given time using a multilabel counter (Wallac 1420 Victor2). The data were corrected based on the controls without macrophages. Quantitative analysis was performed by determination of the areas under the curve (AUC) using the GraphPad Prism software. Furthermore, ${\text{O}}_{2}^{-}$ levels were measured by the nitroblue tetrazolium (NBT, Sigma) reduction assay, which was carried out according to Choi et al. (2006), with minor modifications. Briefly, the macrophages seeded in 24-well plates were supplemented with 1 mg/mL NBT, and infected with the *P. aeruginosa* strains (MOI = 10). After 60 min of infection, intracellular NBT was solubilized and the optical densities were determined spectrophotometrically (Supplementary Material).

The extracellular H_2_O_2_ released from the infected macrophages was measured by the production of Resofurin, using Amplex Red assays (Invitrogen). Briefly, HMDMs were seeded in 96-well plates (10^5^ cells/well) in antibiotic-free culture medium the day before infection, and then washed with PBS and incubated with 50 μM Amplex Red and 0.1 U/mL horse-radish peroxidase in KRPG buffer (145 mM NaCl, 5.7 mM sodium phosphate, 4.86 mM KCl, 0.54 mM CaCl_2_, 1.22 mM MgSO_4_, 5.5 mM glucose, pH 7.35). Macrophages were challenged with *P. aeruginosa* strains at a MOI of 10 and fluorescence was measured at 30 min intervals with a fluorescence scanner (Amersham Typhoon 9600). Values were corrected for controls without macrophages in KRPG supplemented with 50 μM Amplex Red and 0.1 U/mL horse-radish peroxidase. The concentration of H_2_O_2_ in the samples was calculated using standard curves obtained with defined H_2_O_2_ concentrations (0–20 μM).

### Immunoblotting

10^6^ RAW264.7 macrophages were infected with *P. aeruginosa* strains for 1 h, as described above. After infection, macrophages were gently washed and lysed in Hepes 50 mM pH 7.4, NaCl 150 mM, EDTA 20 mM, NaF 100 mM, Na_3_VO_4_ 10 mM, 1% Triton X-100, protease inhibitor cocktail. 25–30 μg protein samples were separated by SDS-PAGE and transferred to nitrocellulose blotting membrane (GE Healthcare, Italy). After blocking in PBS, Tween 0.1%, skim milk 5% (SIGMA, United States), membranes were incubated with the primary antibody for 16–18 h at 4°C. Next day, membranes were washed, incubated at room temperature with the horse-radish-peroxidase–conjugated secondary antibody (GE Healthcare, Italy) and visualized with a Chemi Doc XRS system (Bio-Rad Laboratories Ltd., Hemel Hempstead, United Kingdom). Quantitative Western blotting was performed using the ImageJ software. The primary antibodies used were: LC3 (Cell Signaling, Italy; #2775), diluted at 1:1000; and GAPDH (Santa Cruz, Italy; sc-47724), diluted at 1:400.

### Statistical Analysis

All of the data are reported as a means ± standard deviation. The Figures and statistical analyses were constructed using the GraphPad Prism software (GraphPad Software Inc.). The statistical tests used are indicated in the corresponding Figures. Differences were significant for a *P*-value cut-off of 0.05.

## Results

### SODB Contributes to Short-Term Intracellular *P. aeruginosa* Killing by Macrophages

**FIGURE 1:**
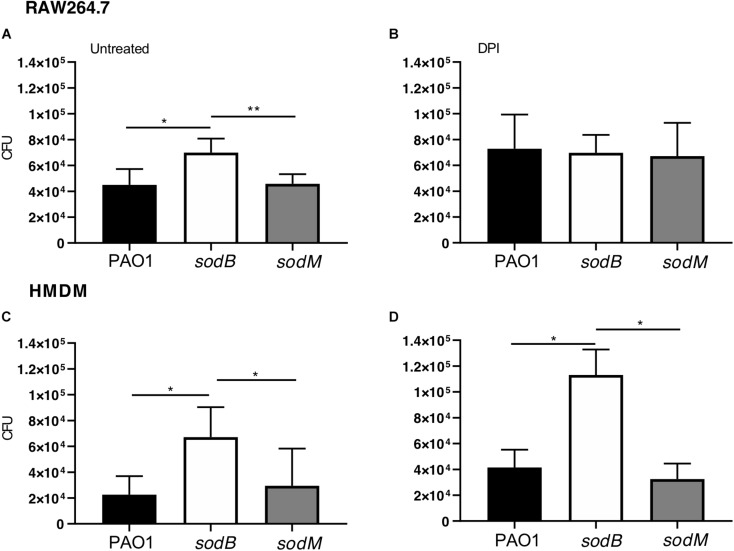
Short-term survival of *Pseudomonas aeruginosa sod* mutants in macrophages. Intracellular survival of *P. aeruginosa* in macrophages infected with PAO1 WT, *sodB*, and *sodM* mutant strains. RAW264.7 macrophages **(A,B)** and HMDMs **(C,D)** were infected with the indicated strains, treated with antibiotics, and lysed with 1% Triton X-100. The total live bacteria recovered on LB medium is reported (CFU). **(B,D)** The cells were pretreated with the NADPH oxidase inhibitor diphenyleneiodonium (DPI; 10 μM for 30 min). Data are means ± standard deviation of six **(A,B)** and three **(C,D)** independent experiments. * *P* < 0.05; ** *P* < 0.01 (Student’s *t*-tests).

The periplasmic *P. aeruginosa* SOD might contribute directly to the scavenging of the superoxide anion (${\text{O}}_{2}^{-}$) produced by the macrophage NOX2, with a possible impact on the bacterial survival. To test this hypothesis, we analyzed the survival of the *P. aeruginosa sod* mutants in the macrophages. For this, RAW264.7 macrophages were infected with WT PAO1 or the *sod* mutants, as either PAO1 *sodB* or s*odM*, and the live intracellular bacteria were determined using the CFU assay. These data showed greater intracellular survival of the PAO1 *sodB* mutant, with respect to the PAO1 WT and PAO1 *sodM*, with these last two showing similar survivals (Figure 1A). As expected, inhibition of NADPH oxidase by the DPI pretreatment resulted in significant increases in the live *P. aeruginosa* recovered from the macrophages infected with PAO1 WT or PAO1 *sodM*, which confirmed the oxidative burst as the primary killing mechanism in the macrophages early after infection (Figure 1B). However, no differences were detected in the PAO1 *sodB* survival in DPI-treated macrophages, with respect to the untreated cells (Figures 1A,B). These data suggested that SODB contributes to the NADPH-dependent killing of *P. aeruginosa* by the macrophages. Similarly, HMDMs showed significant increases in the intracellular survival of PAO1 *sodB*, but not PAO1 *sodM*, with respect to PAO1, in both the untreated and DPI-treated cells (Figures 1C,D). This thus extended the role of *P. aeruginosa* SODB to primary human macrophages.

**FIGURE 2:**
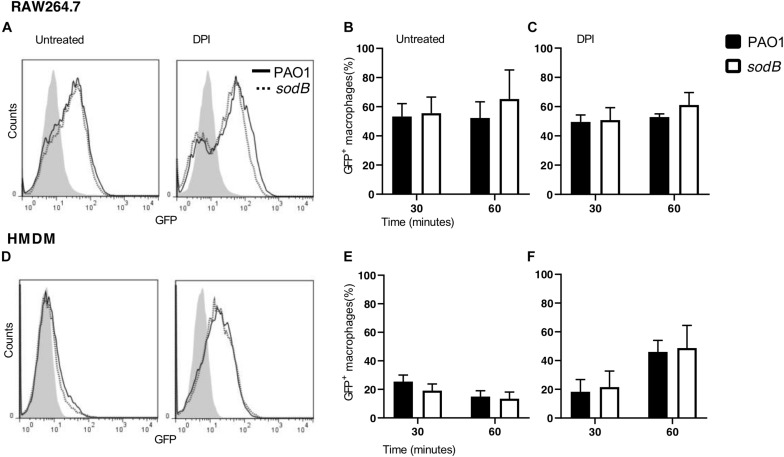
*Pseudomonas aeruginosa* phagocytosis is not affected by SODB. Representative plots of RAW264.7 macrophages **(A)** and HMDMs **(D)**, untreated (left) or treated with the NADPH oxidase inhibitor diphenyleneiodonium (DPI; right) and infected with GFP-expressing PAO1 WT and the *sodB* mutant, as indicated. The proportions of GFP^+^ macrophages at the indicated time of infection was evaluated by flow cytometry **(B,C,E,F)**. Data are means ± standard deviation of three independent experiments. No significant differences were found between PAO1- and *sodB*-infected macrophages (two-way ANOVA; Student’s *t*-tests).

To further support these data, we analyzed phagocytosis by determining the fraction of macrophages that engulfed the PAO1 or PAO1 *sodB* strains. RAW264.7 macrophages and HMDMs were infected with GFP-expressing strains for 30 and 60 min. Subsequently, the non-internalized *P. aeruginosa* were removed by several washes, and the sub-population of infected macrophages (GFP^+^) was evaluated by flow cytometry. Figures 2A,D show no differences in the GFP^+^ sub-population between the cells infected with PAO1 WT and the PAO1 *sodB* mutant, in both the untreated and DPI-treated macrophages. Furthermore, quantitative analysis confirmed that the GFP^+^ sub-populations were similar, irrespective of whether the *P. aeruginosa* infecting strain was PAO1 WT or the PAO1 *sodB* mutant, and whether macrophages were untreated (Figures 2B,E) or DPI-treated (Figures 2C,F). Collectively, these data strongly suggest that the phagocytosis of PAO1 and PAO1 *sodB* was substantially similar at 30 and 60 min after infection.

Having observed greater survival of PAO1 *sodB* with respect to PAO1 WT and no differences in bacterial phagocytosis, we hypothesized that the bacterial SOD, and in particular SODB, contributes to the killing of the intracellular *P. aeruginosa* through the modulation of NADPH-dependent ROS production.

### High ${\text{O}}_{2}^{-}$ and Low H_2_O_2_ Differentiate Macrophages Infected With PAO1 *sodB*

Taking into consideration the enzymatic activities of NADPH oxidase and SOD, the most likely hypothesis to explain these data was that the superoxide radicals produced by the macrophage NOX2 were promptly converted into H_2_O_2_ by the bacterial SOD, which in turn contributes to the killing of the intracellular *P. aeruginosa*. If this is the case, the macrophages infected with the *sod* mutants should be characterized by higher ${\text{O}}_{2}^{-}$ and lower H_2_O_2_ levels than those infected with PAO1 WT. Therefore, we analyzed the ${\text{O}}_{2}^{-}$ and H_2_O_2_ levels in macrophages infected with PAO1 WT and the *sodB* mutant. To differentiate between ${\text{O}}_{2}^{-}$ and H_2_O_2_, we took advantage of their different mobilities through the membrane. Indeed, while the mobility of ${\text{O}}_{2}^{-}$ is highly restricted by its negative charge, as H_2_O_2_ is neutral and long-lived, it can leak from the cells (Fang, 2011). Thus, ${\text{O}}_{2}^{-}$ was measured intracellularly by luminol oxidation (Bedouhène et al., 2017), and H_2_O_2_ extracellularly using Amplex Red assays (Schürmann et al., 2017).

**FIGURE 3:**
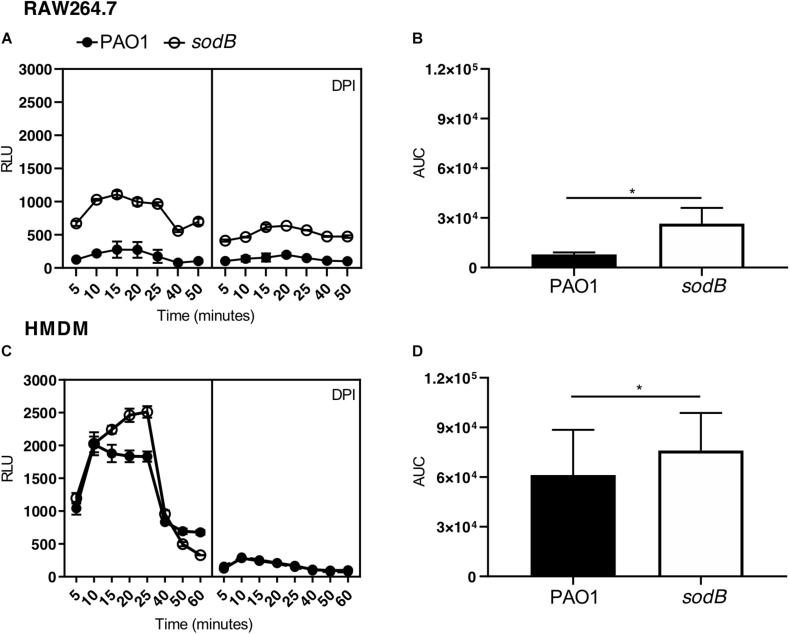
*Pseudomonas aeruginosa* SODB targets the phagocytic ${\text{O}}_{2}^{\text{-}}$. Measurements of superoxide anions using luminol in RAW264.7 macrophages **(A,B)** and HMDMs **(C,D)**. Representative time courses of luminol chemiluminescence in the untreated cells **(A,C**, left panel) and cells pretreated with the NADPH oxidase inhibitor diphenyleneiodonium **(A,C**, right panel) (DPI; 10 μM), and infected with the indicated *P. aeruginosa* strains at a multiplicity of infection of 10. Data are means ± standard deviation of three technical replicates. **(B,D)** Quantification of ${\text{O}}_{2}^{\text{-}}$, as determined by the area under the curve (AUC) from three independent experiments, each carried out in triplicate. RLU, relative luminescent units; * *P* < 0.05 (Student’s *t*-tests).

Macrophages challenged with PAO1 WT and PAO1 *sodB* were loaded with luminol, and ROS production was determined by chemiluminescence measurements. Kinetics analysis revealed a rapid increase in luminol chemiluminescence, which is a readout of ${\text{O}}_{2}^{-}$ production, with peaks at 15 to 25 min (Figure 3). Additionally, the macrophages infected with PAO1 *sodB* showed higher chemiluminescence signals than those infected with PAO1 WT, both for the RAW264.7 cells (Figure 3A) and the HMDMs (Figure 3C). As expected, inhibition of NADPH oxidase with DPI greatly reduced the luminol oxidation (Figures 3A,C). Quantitative analysis of the luminol chemiluminescence AUC confirmed the significantly higher ${\text{O}}_{2}^{-}$ production in the cells infected with PAO1 *sodB*, with respect to PAO1 WT (Figures 3B,D). Similar data were obtained with nitroblue tetrazolium reduction assays, which detected higher levels of the superoxide anion in macrophages infected with the *sodB* mutant, with respect to PAO1 WT (Supplementary Figure S3). Collectively, these data suggest that *P. aeruginosa* SODB contributes to ${\text{O}}_{2}^{-}$ dismutation, and that the extent of its contribution depends on the total amount of ${\text{O}}_{2}^{-}$ produced by the host cells, which, in our experimental models is higher in human than murine macrophages.

**FIGURE 4:**
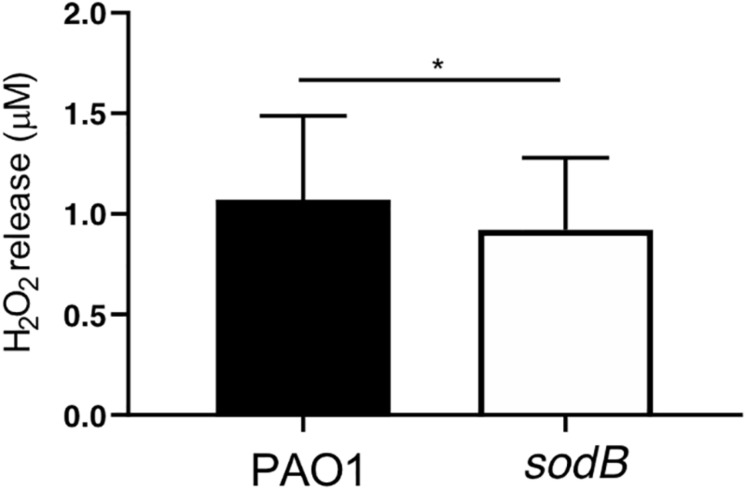
*Pseudomonas aeruginosa* SODB modulates phagocytic ROS. Quantitative analysis of H_2_O_2_ released from HMDMs infected with PAO1 and the *sodB* mutant (as indicated), 90 min after infection. Data are means ± standard deviation of four independent experiments. * *P* < 0.05 (Student’s *t*-tests).

According to our hypothesis, the absence of bacterial SODB activity increases ${\text{O}}_{2}^{-}$ levels and decreases H_2_O_2_ production in macrophages infected with the *sodB* mutant. To confirm this, we evaluated extracellular H_2_O_2_ leakage using the Amplex Red assay. As expected, infection of HMDMs with the *P. aeruginosa sodB* mutant was associated with lower H_2_O_2_ release, with respect to cells infected with PAO1 WT (Figure 4). No signal above the background (i.e., uninfected macrophages) was detected for the RAW264.7 macrophages, possibly due to the low levels of ROS produced upon infection (data not shown).

Overall, the macrophages infected with the PAO1 *sodB* mutants were characterized by higher ${\text{O}}_{2}^{-}$ levels and lower H_2_O_2_ levels, with respect to the cells infected with the WT bacteria, This supported the concept that *P. aeruginosa* SODB converts the phagocytic ${\text{O}}_{2}^{-}$ to H_2_O_2_.

### SODB Promotes Long-Term *P. aeruginosa* Survival in Macrophages

**FIGURE 5:**
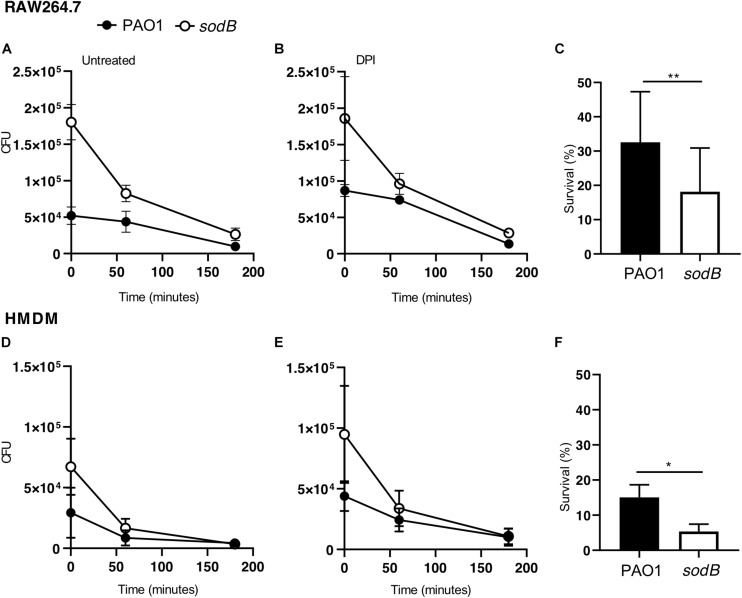
Bacterial SODB enhances long-term intracellular survival within macrophages. Survival curves of *Pseudomonas aeruginosa* in RAW264.7 macrophages **(A,B)** and HMDMs **(D,E)**. The cells were infected with the indicated *P. aeruginosa* strains, and untreated and pretreated with the NADPH oxidase inhibitor diphenyleneiodonium (DPI; 10 μM for 30 min), as indicated. At the end of the infection macrophages (time 0) and after 60 and 180 min, samples were collected and the CFU determined. **(C,F)** Bacterial survival in untreated RAW264.7 **(C)** and HMDMs **(F)** at 180 min from the end of infection. Data are means ± standard deviation of three independent experiments. * *P* < 0.05; ** *P* < 0.01 (Student’s *t*-tests).

It has been well established that macrophages can kill engulfed bacteria using different mechanisms that can be activated sequentially, starting with the oxidative burst, which in turn activates other mechanisms, including autophagy (Huang et al., 2009; Lam et al., 2010). Thus, to evaluate the possible role of the bacterial SODB in microbicidal mechanisms other than the oxidative burst, we analyzed *P. aeruginosa* survival at later time points after infection. After infection and treatment with antibiotics, the RAW264.7 macrophages were incubated in bacteria-free medium for 1 h and 3 h, and the live intracellular bacteria were counted using the CFU assay (Figures 5A–C). As previously observed, at the end of the infection (*t*_0_), more viable PAO1 *sodB* were recovered from the infected macrophages than the PAO1 WT (Figures 5A,B, time 0). However, the CFU recovered from the infected macrophages at 1 h and 3 h from the infection were similar, independent of the bacterial strains (Figures 5A,B). Similar data were obtained in the HMDMs (Figures 5D,E). Consequently, the long-term survival of PAO1 WT appeared to be greater than that of PAO1 *sodB* in both of these macrophage models (Figures 5C,F). This was also described by the slopes of the killing curves, which represent the rates of bacterial killing, and which were higher for PAO1 *sodB*, with respect to PAO1 WT (Supplementary Table S2).

Collectively, these data demonstrate that the long-term survival of the PAO1 *sodB* mutant in both murine and human macrophages is less than for PAO1 WT, which suggests that SODB ultimately promotes bacteria survival.

### SODB Contributes to Decreased Autophagy Activation in Macrophages

**FIGURE 6:**
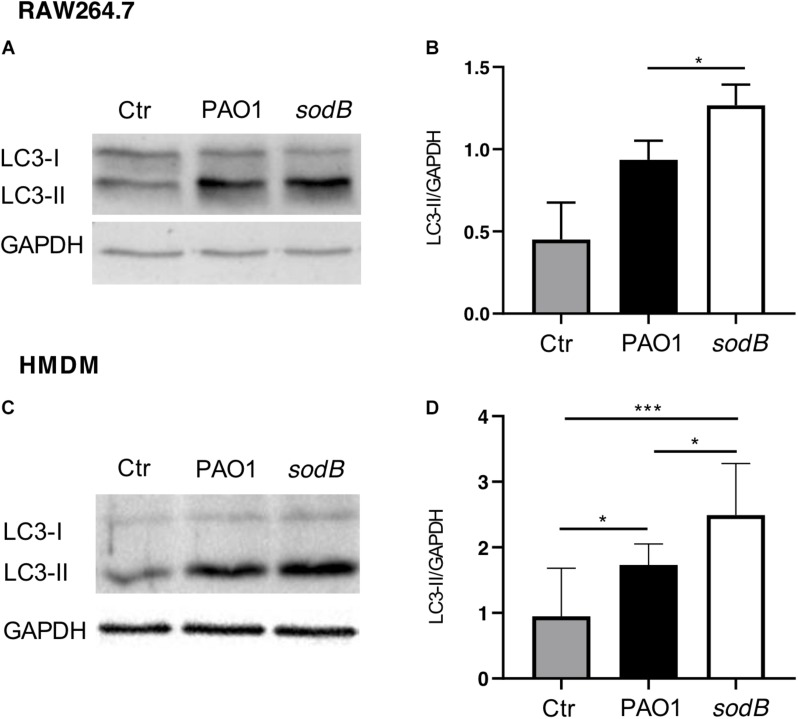
*Pseudomonas aeruginosa* SODB reduces autophagy activation in macrophages. RAW264.7 macrophages **(A,B)** and HMDMs **(C,D)** were infected with the indicated *P. aeruginosa* strains. After infection, whole cell lysates were immunoblotted with anti-LC3 and anti-GAPDH (loading control) antibodies. **(A,C)** Representative Western blots. **(B,D)** Quantitative analysis of band intensities, reported as the LC3-II/GAPDH ratio. Data are means ± standard deviation of three independent experiments. Ctr, uninfected macrophages. * *P* < 0.05; *** *P* < 0.001 (Student’s *t*-tests).

It has been shown that autophagy activation by Toll-like receptor signaling or receptor-mediated phagocytosis depends on NOX2 activity and ROS production, with the latter required to recruit LC3 to phagosomes and to target intracellular *Salmonella typhimurium* to (auto)phagosomes (Huang et al., 2009). Additionally, autophagy activation by *P. aeruginosa* has been demonstrated in different cell types, including macrophages, where it contributes to restrict intracellular survival (Yuan et al., 2012; Junkins et al., 2013; Jabir et al., 2014). Having observed that *P. aeruginosa* SODB contributed to the long-term survival of *P. aeruginosa* within macrophages, we speculated that it might inhibit autophagy through reduction of the levels of intracellular ${\text{O}}_{2}^{-}$. To test this hypothesis, RAW264.7 cells and HMDM macrophages were infected with PAO1 WT and PAO1 *sodB*, and autophagy activation was monitored by analysis of changes in the levels of lipidated LC3 (LC3-II) with Western blotting (Kabeya et al., 2000). In whole-cell lysates from these non-infected macrophages, LC3-I and LC3-II were detected at similar levels in the RAW264.7 cells, while in HMDMs the intensity of the LC3-II band was slightly higher than that of LC3-I. In contrast, 1 h after infection, LC3-II increased specifically in the infected macrophages (Figures 6A,C). Accordingly, quantitative Western blotting showed higher levels of LC3-II in cells infected with the PAO1 *sodB* mutant, with respect to those infected with PAO1 WT (Figures 6B,D). This suggests that the SODB activity of the intracellular *P. aeruginosa* reduces autophagy activation, which thus provides a link between the oxidative and non-oxidative roles of SODB in modulation of *P. aeruginosa* survival in macrophages.

## Discussion

Macrophages are key players in the innate immune system, due to their engulfing of pathogens and activation of an arsenal of antimicrobial weapons. However, whether macrophages successfully accomplish this task is also dependent on microbial responses to the microbicidal mechanisms. NADPH oxidase activation is an early response to infection by macrophages, and this contributes to killing of intracellular bacteria and activation of other microbicidal mechanisms, such as autophagy. Consequently, ROS scavengers should be a determinant for the engulfed bacteria to survive the hostile intracellular environment, both in the immediate phase and in the later phases of infection. *P. aeruginosa* has two SOD genes, *sodB* and *sodM*, which encode the iron- and manganese-cofactored SODs, respectively, both of which have been identified as periplasmic enzymes (Yu et al., 2010). These SODs are part of the bacterial defenses that target the different forms of ROS and transform them into less toxic compounds. The periplasmic localization of the SODs is strategic, as this allows targeting of the superoxide radicals (${\text{O}}_{2}^{-}$), which might enter this compartment from the environment. For bacteria enclosed in phagosomes, the superoxide radicals produced by NADPH-oxidase are likely to be rapidly transferred into the periplasm, both facilitated by their protonation to HO_2_, and aided by the V-ATPase that pumps H^+^ within the phagosomal lumen (Burton et al., 2014). In the bacterial periplasm, the HO_2_ can be targeted by SODs and dismutated to H_2_O_2_. The resulting H_2_O_2_ can be further processed by other bacterial scavenger enzymes, catalases, and peroxidases, although it might also contribute to bacterial killing, depending on the balance between the oxidative stress imposed by the host cell and the scavenger activities of the engulfed bacteria. Here, we have shown that macrophages infected with PAO1 *sodB* are differentiated by increased levels of ${\text{O}}_{2}^{-}$ and lower levels of H_2_O_2_, with respect to those infected with PAO1 WT. This turns out to have a negative impact on bacterial survival within the macrophages, as we found that the short-term survival of the PAO1 *sodB* mutant was greater than that of PAO1 WT. Taken together, these data allow us to speculate that for the bacterial cells, periplasmic ${\text{O}}_{2}^{-}$ is less toxic than H_2_O_2_, possibly because few, if any, ${\text{O}}_{2}^{-}$ targets are present in this compartment, and/or because of the high mobility of H_2_O_2_, which allows its entry into the cytoplasm, which contains the most vulnerable ROS targets. Furthermore, as SOD restricts *P. aeruginosa* survival within macrophages, we identified this activity as pro-microbicidal for the bacterial cells, rather than pro-survival. From another point of view, it can be speculated that macrophages improve their microbicidal activity using the bacterial SOD activity. Of the two *P. aeruginosa* SODs, we show here that only SODB has a role in intracellular survival, which suggests that the iron-cofactor is not limiting in this environment, and that SODB is the major enzyme that acts within phagocytes. This finding is in agreement with previous studies that were also based on the analysis of SOD activities in clinical isolates, which indicates that SODB is more important than SODM in the protection of *P. aeruginosa* from oxidative stress (Hassett et al., 1995; Britigan et al., 2001). Interestingly, a lung *P. aeruginosa* isolate that lacks SODM activity has been reported (Polack et al., 1996).

The SODB pro-microbicidal activity appears to be difficult to reconcile with the role of this gene in mediating *P. aeruginosa* virulence (Hassett et al., 1995; Britigan et al., 2001; Iiyama et al., 2007). Furthermore, considering that we have analyzed the survival of intracellular *P. aeruginosa* and that macrophages show different microbicidal mechanisms, it is reasonable to surmise that SODB activity might ultimately be pro-survival. Indeed, as macrophages trigger the different microbicidal mechanisms in a time-dependent manner, and as some of these are activated by ROS, we have reasoned that *P. aeruginosa* modulation of phagocytic ROS might have an impact on the activation of non-oxidative microbicidal mechanisms. Our data here show that 3 h after infection, the survival of PAO1 *sodB* was reduced, with respect to PAO1 WT, which suggests that SODB activity indirectly promotes long-term bacterial survival. As microbicidal mechanisms are activated stepwise after phagocytosis, with the oxidative burst being rapidly exhausted (Figures 3A,C), we reasoned that non-oxidative, but ROS dependent, microbicidal mechanisms might be modulated by the bacterial SODB. Accordingly, we observed that macrophages infected with the PAO1 *sodB* mutant showed stronger activation of autophagy, as assessed by LC3 lipidation, which suggests that SODB inhibits autophagy activation. This is possible, as SODB changes the levels of the different ROS formed within the *P. aeruginosa*-infected phagosomes in favor of H_2_O_2_, which might be further detoxified or be removed from the host cells. By doing so, the engulfed bacteria consume NOX2-dependent superoxide radicals, and concomitantly, reduce the signal for autophagy activation, and hence for further bacterial killing. In brief, while SODB initially imposes higher oxidative stress on the bacteria within the phagosome, by reducing the ${\text{O}}_{2}^{-}$ levels later on, it contributes to the dampening of the autophagy activation. This might directly target bacteria enclosed in the LC3 Associated Phagosome (LAP) or bacteria that escape the phagosome and are intercepted in the cytosol by the autophagic flux. In favor of the latter mechanism, it was recently reported that *P. aeruginosa* first resides in the phagosome, and then following phagosome rupture, it can be detected in the cytosol (Garai et al., 2019).

**FIGURE 7:**
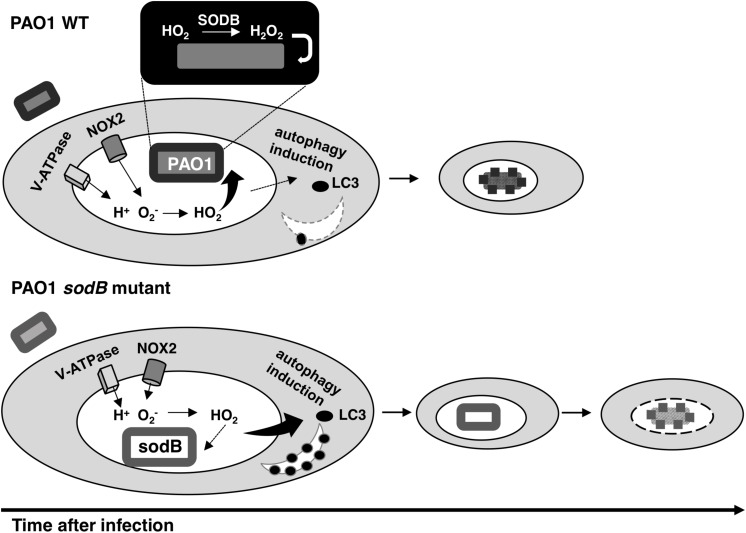
Model for the role of SODB in *Pseudomonas aeruginosa* killing by macrophages. The phagocytic NADPH oxidase can pump superoxide radicals (${\text{O}}_{2}^{\text{-}}$) into the phagosome lumen. Here, the protonated form of ${\text{O}}_{2}^{\text{-}}$ (HO_2_) can pass into the engulfed bacteria or the host cytosol. When the phagosome captures a bacterium with SODB activity (PAO1 WT), the HO_2_ within the periplasm is dismutated to H_2_O_2_ and the bacterium is killed. Consequently, SODB depletes the superoxide radicals from the phagosome. In the absence of SODB activity (PAO1 *sodB* mutant), the HO_2_ is mainly transferred to the host cytosol, where it promotes autophagy induction, which in turn improves bacterial killing at the later stages following infection.

Overall, our data are summarized in the model depicted in Figure 7, which considers the pro-microbicidal and pro-survival activities of *P. aeruginosa* SODB within macrophages. Importantly, our model is supported by these data obtained with both murine and human macrophages, although these two cellular models differ in the ${\text{O}}_{2}^{-}$ levels produced by these macrophages in response to bacterial infections.

## Data Availability Statement

All datasets generated for this study are included in the article/Supplementary Material.

## Ethics Statement

The studies involving human participants were reviewed and approved by local ethics committee (Comitato Etico, Azienda Policlinico Umberto I, Rome, Italy; 1233/2016). The patients/participants provided their written informed consent to participate in this study.

## Author Contributions

FA and PD conceptualized and defined the experimental design. LC, EG, and FL performed the experiments. PD and ED contributed materials and analysis tools. LC and FA carried out the data analysis and Figure preparation, and wrote the manuscript.

## Conflict of Interest

The authors declare that the research was conducted in the absence of any commercial or financial relationships that could be construed as a potential conflict of interest.
