# Supplementary material for: Escaping the Phagocytic Oxidative Burst: The Role of SODB in the Survival of Pseudomonas aeruginosa Within Macrophages
Source: Front Microbiol. 2020 Mar 10;11:326. doi: 10.3389/fmicb.2020.00326 (PMC7077434; doi:10.3389/fmicb.2020.00326)
Supplement: Supplementary file 1 [file Data_Sheet_1.docx]

**Supplementary data**

**Analysis of *sodB* and *sodM* mutant strains by PCR**

Samples recovered from frozen stocks were streaked onto selective plates (LB agar containing 50 μg/mL tetracycline or gentamycin for sodB and sodM, respectively) and incubated overnight at 30°C. Next, the presence of the SodB and SodM genes was determined by PCR in two single-colony subclones. The PCR primers are listed in Table 1. PCR reactions were carried out under standard conditions with minor modifications: 10 μg/mL bovine serum albumin was added to the reactions. The pre-denaturation cycle was performed at 95 °C for 10 min; this followed 35 cycles, each including the following steps: 95 °C for 30 s, 60 °C for 30 s, 72 °C for 30 s; and finally, 1 cycle at 72 °C for 7 min.

As expected, the SodB gene band was present in the *sodM* mutant strain but absent in the *sodB* mutant strain, while the contrary was observed for the sodM band, which was present in the *sodB* mutant strain but absent in the *sodM* mutant strain; both the sodB and sodM bands were present in the parental PAO1 WT strain (**Supplementary Figure S1**).

***P. aerugionosa* growth in LB and RPMI media**

It is known that *P. aeruginosa* *sodB* and *sodB*, *sodM* double mutants grow slowly, with the latter showing the more severe defect (Hasset et al., 1995; Iiyama et al., 2007). In contrast, the *sodM* mutant does not show any growth defect (Hasset et al., 1995; Iiyama et al., 2007). To confirm this, the growth of the *P. aeruginosa* strains was analyzed in LB broth at 30 °C, as previously reported (Iiyama et al., 2007). Typically, stationary phase bacteria were diluted in pre-warmed LB medium, and grown to mid-exponential phase (OD_600_, 0.3-0.4). As expected, the growth of the *sodB* mutant was slower than that of the parental PAO1 (**Supplementary Figure S2**), while no difference was seen between PAO1 WT and *sodM* mutant (data not shown). Furthermore, as the macrophage infection assays were performed in RPMI medium at 37 °C with 5% CO_2_, the growth of the *P. aeruginosa* strains were also monitored under these conditions. Here, the growth of *P. aeruginosa* was very slow, with no difference between the two strains (**Supplementary Figure S2**).

**ROS measurement using the NBT assay**

The nitroblue tetrazolium (NBT, Sigma) colorimetric reduction assay, has been reported to selectively detect O_2_^-^ (reviewed in Zielonka et al., 2017). Therefore, NBT was used to support data obtained with luminol. The NBT assay was essentially carried out according to Choi (2006), with minor modifications. Briefly, macrophages were seeded in 24-well plates at 3 ×10⁵ cells/well, supplemented with 1 mg/mL NBT, and infected with the *P. aeruginosa* strains at a multiplicity of infection of 10, or stimulated with 400 ng/mL phorbol 12-myristate 13-acetate (PMA). Where necessary, the cells were pretreated with 10 μM DPI for 30 min. After 60 min of infection, the cells were washed three times with phosphate-buffered saline and the intracellular NBT was solubilized by addition of 160 μL 2 M KOH and 100 μL dimethylsulfoxide to each well. The optical densities were determined spectrophotometrically at 620 nm (Ultrospec II; LKB Biochrom).

First, the NBT assays were set up using macrophages stimulated with PMA. Increase in OD_620_ were seen for the PMA-stimulated cells, with respect to unstimulated cells, although only the increase for HMDMs reached statistical significance. Next, the assay was used to measure ROS production in macrophages infected with PAO1 WT and the *sodB* mutant, 1 h after infection. These data showed higher levels of the superoxide anions in macrophages infected with the *sodB* mutant, with respect to PAO1 WT, in both the murine (RAW264.7 macrophages) and human (HMDMs) cells (**Supplementary Figure S3**). No signal above the background (i.e., uninfected cells) was detected in cells pretreated with the NADPH oxidase inhibitor diphenyleneiodonium (DPI; 10 μM for 30 min) (data not shown).

**Supplementary Figures**

**
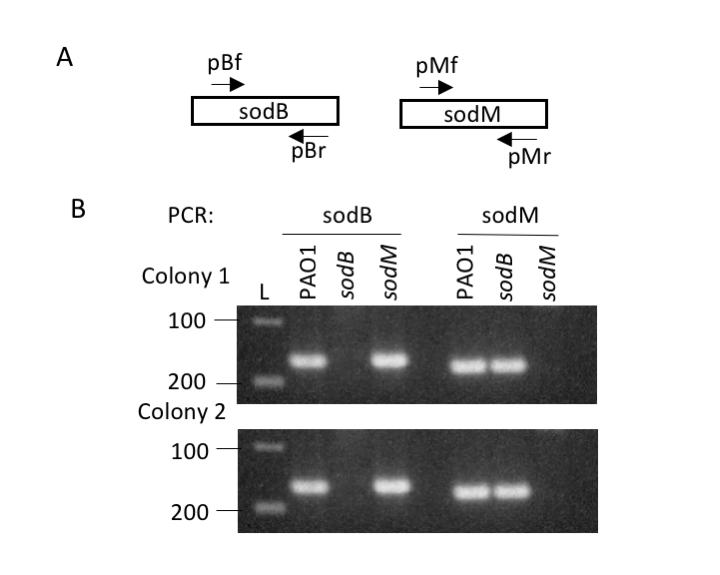
**

**Supplementary Figure S1. The *sodB* and *sodM* mutant strains show the expected gene deletions.** **(A)** Schematic representation of the PCR reaction. **(B)** SodB and SodM amplification bands in the indicated PAO1 WT and *sodB* and *sodM* mutant strains, with two single-colony cultures (colony 1, colony 2) reported for each strain. The PCR reactions were analyzed using agarose gel electrophoresis (1.5% agarose gels; TBE 0.5× buffer). L, molecular weight marker. Band sizes are given in bp.


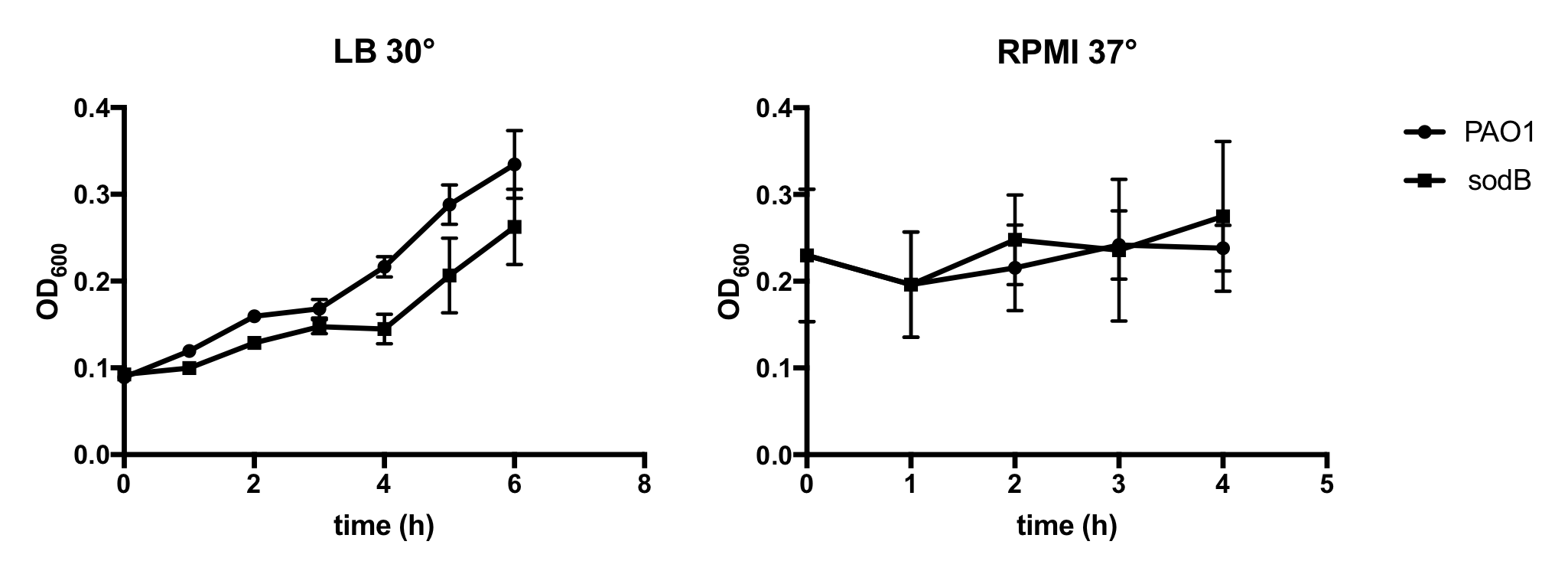


**Supplementary Figure S2. Growth kinetics of Pseudomonas *aeruginosa* in LB medium at 30 °C (left) and in RPMI at 37 °C, with 5 % CO_2_ (right).** Data are means ±standard deviation of three independent experiments.


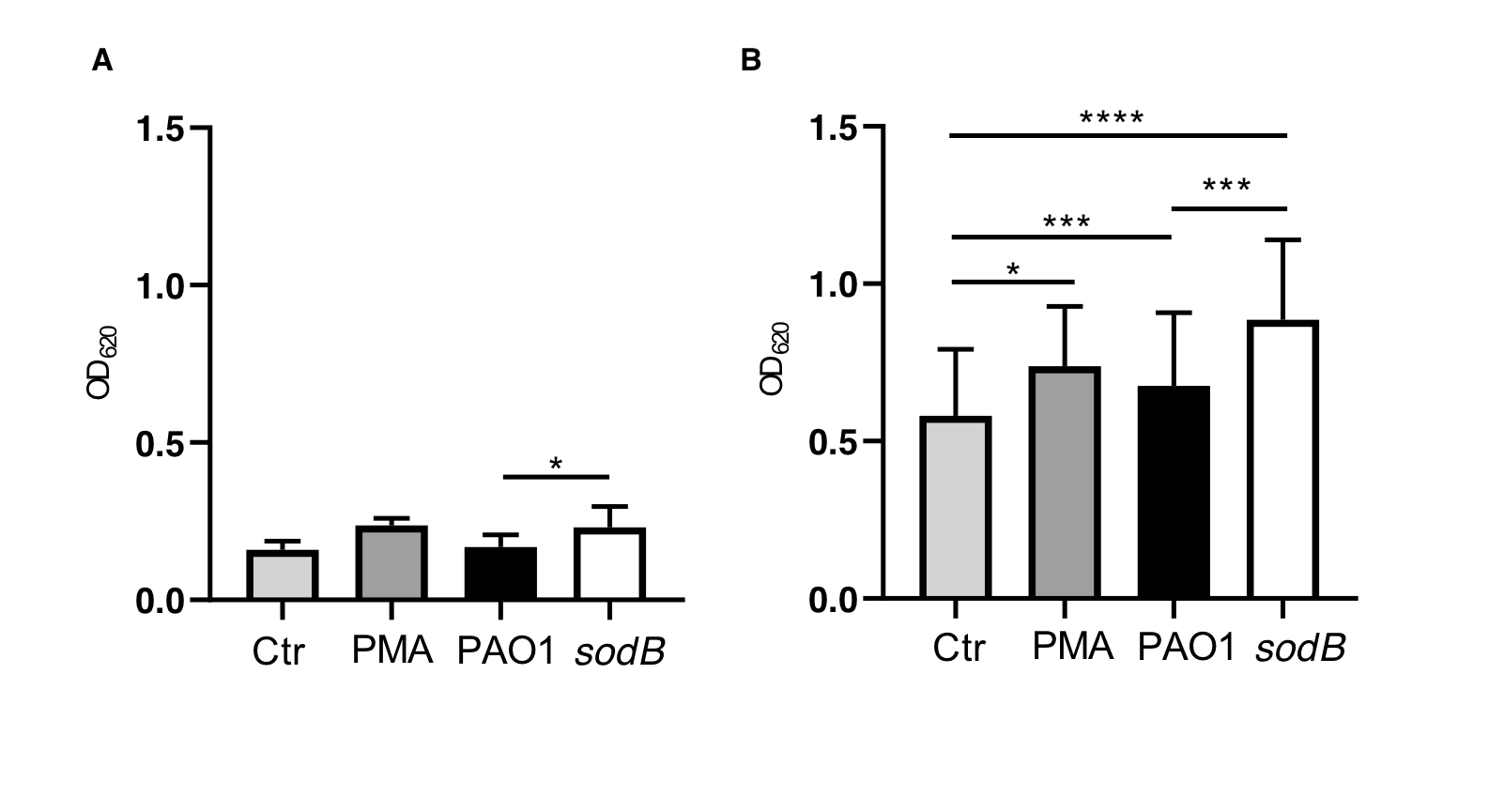


**Supplementary Figure S3. O_2_^-^ detection using the NBT assay in infected macrophages.** Colorimetric O_2_^-^ detection in the RAW264.7 macrophages **(A)** and HMDMs **(B)** infected with the indicated strains or treated with phorbol 12-myristate 13-acetate (PMA). Ctr, uninfected macrophages. Data are means ±standard deviation (n = 3). *, *P* <0.05; **, *P* <0.01; ****P* <0.005; *****P*<0.0001 (Student’s *t*-tests).

**Supplementary Table S1**. Primer used in this study.

| **Target gene** | **Primer** | **Sequence (5’-3’)** | **Amplicon size** |
| --- | --- | --- | --- |
| SodB | pBf | CGAAAAGAACGCCCTTGAGC | 126 bp |
|  | pBr | CTCTTGCCTTCGAACTCGGT |  |
| SodM | pMf | GTTGCCGCAGCAGACTTTC | 114 bp |
|  | pMr | TGACCATGGAGATCCACCAC |  |

**Supplementary Table S2.** Slope data of the survival curves. *

|  | **wo** | | **DPI** | |
| --- | --- | --- | --- | --- |
|  | **PAO1** | **sodB** | **PAO1** | **sodB** |
| RAW264.6 cells | -242.4 | -798.3 | -421.0 | -828.6 |
| HMDMs | -124.7 | -318.9 | -178.5 | -426.9 |

*Data refer to the survival curves reported in Figure 5 of the main manuscript

**References cited in Supplementary Materials**

Choi HS, Kim JW, Cha YN, Kim C. A quantitative nitroblue tetrazolium assay for determining intracellular superoxide anion production in phagocytic cells. J Immunoassay Immunochem. 2006;27(1):31-44.

Hassett DJ, Schweizer HP, Ohman DE. *Pseudomonas aeruginosa* sodA and sodB mutants defective in manganese- and iron-cofactored superoxide dismutase activity demonstrate the importance of the iron-cofactored form in aerobic metabolism. J Bacteriol. 1995;177: 6330–6337. doi:10.1128/jb.177.22.6330-6337.1995

Hassett DJ, Woodruff WA, Wozniak DJ, Vasil ML, Cohen MS, Ohman DE. Cloning and characterization of the *Pseudomonas aeruginosa* sodA and sodB genes encoding manganese- and iron-cofactored superoxide dismutase: demonstration of increased manganese superoxide dismutase activity in alginate-producing bacteria. J Bacteriol. 1993;175: 7658–7665. doi:10.1128/jb.175.23.7658-7665.1993

Iiyama K, Chieda Y, Lee JM, Kusakabe T, Yasunaga-Aoki C, Shimizu S. Effect of superoxide dismutase gene inactivation on virulence of *Pseudomonas aeruginosa* PAO1 toward the silkworm, *Bombyx mori*. Appl Environ Microbiol. 2007; 73: 1569–1575. Doi:10.1128/AEM.00981-0627.

Zielonka J, Hardy M, Michalski R, Sikora A, Zielonka M, Cheng G, et al. Recent developments in the probes and assays for measurement of the activity of NADPH oxidases. Cell Biochem Biophys. 2017; 75(3-4): 335–349. Doi:10.1007/s12013-017-0813-6.
